# Community case management of childhood illness in sub–Saharan Africa – findings from a cross–sectional survey on policy and implementation

**DOI:** 10.7189/jogh.04.020401

**Published:** 2014-12

**Authors:** Kumanan Rasanathan, Maria Muñiz, Salina Bakshi, Meghan Kumar, Agnes Solano, Wanjiku Kariuki, Asha George, Mariame Sylla, Rory Nefdt, Mark Young, Theresa Diaz

**Affiliations:** 1 UNICEF, New York, NY, USA; 2 UNICEF Eastern and Southern Africa Regional Office, Nairobi, Kenya; 3 UNICEF West and Central Africa Regional Office, Dakar, Senegal; 4 Johns Hopkins Bloomberg School of Public Health, Baltimore, MD, USA

## Abstract

**Background:**

Community case management (CCM) involves training, supporting, and supplying community health workers (CHWs) to assess, classify and manage sick children with limited access to care at health facilities, in their communities. This paper aims to provide an overview of the status in 2013 of CCM policy and implementation in sub–Saharan African countries.

**Methods:**

We undertook a cross–sectional, descriptive, quantitative survey amongst technical officers in Ministries of Health and UNICEF offices in 2013. The survey aim was to describe CCM policy and implementation in 45 countries in sub–Saharan Africa, focusing on: CHW profile, CHW activities, and financing.

**Results:**

42 countries responded. 35 countries in sub–Saharan Africa reported implementing CCM for diarrhoea, 33 for malaria, 28 for pneumonia, 6 for neonatal sepsis, 31 for malnutrition and 28 for integrated CCM (treatment of 3 conditions: diarrhoea, malaria and pneumonia) – an increase since 2010. In 27 countries, volunteers were providing CCM, compared to 14 countries with paid CHWs. User fees persisted for CCM in 6 countries and mark–ups on commodities in 10 countries. Most countries had a national policy, memo or written guidelines for CCM implementation for diarrhoea, malaria and pneumonia, with 20 countries having this for neonatal sepsis. Most countries plan gradual expansion of CCM but many countries’ plans were dependent on development partners. A large group of countries had no plans for CCM for neonatal sepsis.

**Conclusion:**

28 countries in sub–Saharan Africa now report implementing CCM for pneumonia, diarrhoea and malaria, or “iCCM”. Most countries have developed some sort of written basis for CCM activities, yet the scale of implementation varies widely, so a focus on implementation is now required, including monitoring and evaluation of performance, quality and impact. There is also scope for expansion for newborn care. Key issues include financing and sustainability (with development partners still providing most funding), gaps in data on CCM activities, and the persistence of user fees and mark–ups in several countries. National health management information systems should also incorporate CCM activities.

Community case management (CCM) involves training, supporting, and supplying community health workers to assess, classify and manage sick children with limited access to care at health facilities, in their communities [1]. In this context, a “community health worker” (CHW) is a health worker delivering health care in the community, trained in some way in the context of the intervention, and having no formal health professional or paraprofessional certificate or tertiary education degree; regardless of whether or not they receive monetary payment. In recent years, there has been increasing momentum for CHWs to provide CCM to prevent mortality and morbidity for pneumonia, malaria, diarrhoea, malnutrition and neonatal infections [2], reflecting the fact that these conditions remain the leading causes of mortality for children under five [3].

Despite the existence of cost–effective and appropriate treatment for these conditions, effective care is often limited due to challenges with access to health facilities, supply of commodities and trained staff, and knowledge and incentives within communities to utilize services in a timely manner [4]. For instance, in sub–Saharan Africa, only 31% of children with diarrhoea receive treatment with oral rehydration salts [5]. Similarly, only 37% of children with fever receive any antimalarial (notwithstanding that not all of these children will have malaria), and medical care was sought for only 46% of children with symptoms of pneumonia [5].

CCM (or integrated CCM, or “iCCM”, where services for diagnosis and treatment for pneumonia, diarrhoea and malaria are provided together) is a strategy that attempts to overcome these deficits by providing support for health care services in the community, close to where people live, complementing, and referring to, facility–based services. Key aspects of CCM programmes include training and support of community health workers (CHWs) and algorithms for community–based treatments of childhood illnesses, such as diarrhoea, malaria, and pneumonia. There is increasing evidence that CCM and CHW programmes can contribute overall to a reduction in child mortality [6–9]. In a 2010 survey of countries in sub–Saharan Africa, 29/40 countries reported implementing CCM for diarrhoea, 26/39 for malaria, and 21/40 for diarrhea [10,11].

This paper aims to provide an overview of the status in 2013 of community case management (CCM) policy and implementation for malaria, pneumonia, diarrhoea, neonatal sepsis and malnutrition for under–5–year–old children in all sub–Saharan African countries. It presents findings from a 2013 cross–sectional, quantitative survey, building on previous surveys of CCM policy and implementation [10,12]. It should be noted that this overview includes both implementation of iCCM for the above conditions as well as CCM programmes for individual conditions that are not integrated. Following this overview paper, future papers are planned to report in–depth data from specific areas of the survey.

## METHODS

We undertook a cross–sectional, descriptive, quantitative survey from August 2013 to January 2014, focusing on community health workers who provide CCM services – that is basic health care services and referral where necessary for malaria, pneumonia, diarrhoea, neonatal sepsis and/or malnutrition for children under 5 years. For the purposes of this survey, CCM for pneumonia refers to at least the delivery by CHWs of an oral antibiotic (amoxicillin or cotrimoxazole); CCM for diarrhoea refers to at least the delivery by CHWs of oral rehydration salts and zinc; CCM for malaria refers to at least the delivery by CHWs of artemisinin–based combination therapy; CCM for neonatal sepsis refers to delivery by CHWs of oral or injectable antibiotics; and CCM for malnutrition refers to screening and referral by CHWs of severe malnutrition.

The survey instrument drew from previous survey studies of CCM implementation and policy to facilitate the possibility of comparison, in particular a survey undertaken in 2010 [10]. The 2013 survey also included questions on a number of areas that had not previously been examined such as monitoring and reporting, and financing. The 2013 survey examined five domains within CCM programming: policy, implementation, CHW profile, CHW activities, and financing. The survey was designed to be completed collaboratively by the focal point for CCM in the respective Ministry of Health for each country along with the technical officer responsible for CCM in each country’s UNICEF office.

The survey was distributed by email to two regional focal points in UNICEF’s Eastern and Southern Africa Regional Office (ESARO) and West and Central Africa Regional Office (WCARO) in Africa, who then distributed the questionnaires by email to focal points for CCM in both Ministries of Health and UNICEF country offices in all 45 countries in sub–Saharan Africa where UNICEF has a country office (see **Table 1**). The in–country focal points were then responsible for liaising with other in–country officials to fill in the questionnaire electronically, and then submit it back to the regional focal points. Where there was no focal point in the Ministry of Health for CCM, the UNICEF country office referred the survey to the most appropriate official. All the completed surveys were received by November 2013. Data entry was conducted by the regional offices using web entry forms designed in Formhub (https://formhub.org/). Triangulation, data cleaning and verification took place between November 2013 and March 2014. Triangulation was undertaken by review of surveys by technical experts in the region, and seeking of clarification on queries from those who originally completed the survey.

**Table 1:** Countries included in the survey

Angola	Liberia
Bénin	Madagascar
Botswana	Malawi
Burkina Faso	Mali
Burundi	Mauritania
Cameroon	Mozambique
Cabo Verde	Namibia
Central African Republic	Niger
Chad	Nigeria
Comoros	Rwanda
Congo	Săo Tomé e Príncipe
Côte d'Ivoire	Senegal
Democratic Republic of Congo	Sierra Leone
Equatorial Guinea	Somalia
Eritrea	South Africa
Ethiopia	South Sudan
Gabon	Swaziland
Gambia	Tanzania
Ghana	Togo
Guinea	Uganda
Guinea–Bissau	Zambia
Kenya	Zimbabwe
Lesotho	

The forms were entered online by two individuals, one from WCARO, and one from ESARO. The data entry screen facilitated: quality control checks and information to be accessed by UNICEF regional and headquarters offices in real–time; data cleaning and tracking of progress of entry; and data backup and sharing between the two offices. Following the entry of the information, the data was exported from Formhub as a csv, and then processed in SPSS (v22). Data processing included cleaning, labelling and transformation of the data.

The following checks were undertaken with the data: 1) missing values; 2) consistency of responses, and 3) range checks. During the data cleaning process, missing questions or questions that needed further clarification were flagged, compiled and submitted to the UNICEF regional offices for follow up with survey respondents. Additionally, country profiles were developed using each country's survey response and were circulated for review, to further assist in the data cleaning process. Finally, provisional results were presented and discussed at the iCCM Evidence Review Symposium in Accra, Ghana, in March 2014, providing further feedback and checks on the data. Descriptive analysis was conducted using Excel and STATA v. 13.0 (StataCorp LP, College Station, Texas, USA).

## RESULTS

Out of the 45 countries surveyed, 42 countries submitted a response. The non–responding countries were Cabo Verde, Săo Tomé e Príncipe and Equatorial Guinea, which do not have CCM programmes, and thus opted out of completing of the survey.

**Figure 1:**
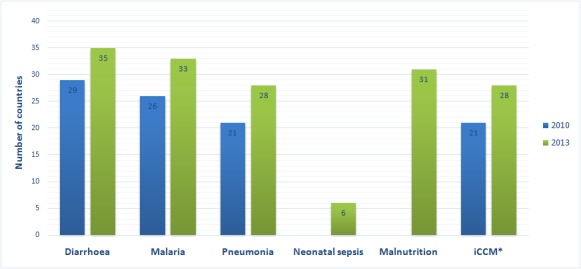
Implementation of community case management (CCM) of diarrhoea, malaria, pneumonia, neonatal sepsis and malnutrition in sub–Saharan Africa (n = 42). iCCM* refers to community case management services for diagnosis and treatment of pneumonia, diarrhoea and malaria that are provided together. There was no data for neonatal sepsis and malnutrition in the 2010 survey.

In 2013, 35 countries out of the 42 countries in sub–Saharan Africa that completed the survey reported implementing CCM for diarrhoea, 33 countries for malaria, 28 for pneumonia, 6 for neonatal sepsis, 31 for malnutrition and 28 countries for iCCM (treatment of 3 conditions: diarrhoea, malaria and pneumonia). There has been an increase in the number of countries implementing CCM for these conditions since 2010, as shown in **Figure 1**. Of the 28 countries implementing CCM for pneumonia, diarrhoea and malaria, 11 were in Eastern and Southern Africa, and 17 were in West and Central Africa. The 6 countries reporting implementation of CCM for neonatal sepsis were Gambia, Ghana, Niger, Democratic Republic of Congo, Swaziland, and Uganda. In all of these countries, implementation has not reached all intended communities and districts with the full range of planned activities. There were 6 countries who responded to the survey who are not implementing CCM activities: Botswana, Gabon, Guinea–Bissau, South Africa, Tanzania, and Zimbabwe.

### Gender and type of CHW and incentives for provision of CCM

The cadres of CHWs providing CCM services were of mixed gender in 17 countries, mostly female in 8 countries, mostly male in 9 countries, and exclusively female in 1 country.

In 27 countries, volunteers were providing CCM, compared to 14 countries where paid CHWs were doing so. Traditional birth attendants were implementing CCM in 3 countries and mid–level providers were doing so in 4 countries. In 5 countries, another type of CHW was providing CCM. It should be noted that in several countries, there was more than one type of cadre providing CCM.

Incentives for CHWs providing CCM varied, with different types of incentives often being used in the same country. A salary was provided by the Ministry of Health in 6 countries, and by non–governmental organizations (NGOs) in 2 countries. User fees were still charged for CCM in 6 countries and mark–ups on commodities in 10 countries, mostly in West Africa. Incentive payments were provided by the Ministry of Health in 10 countries, and by NGOs in 19 countries. In 23 countries, non–monetary incentives were used.

### CCM Policy and national guidelines

**Figure 2:**
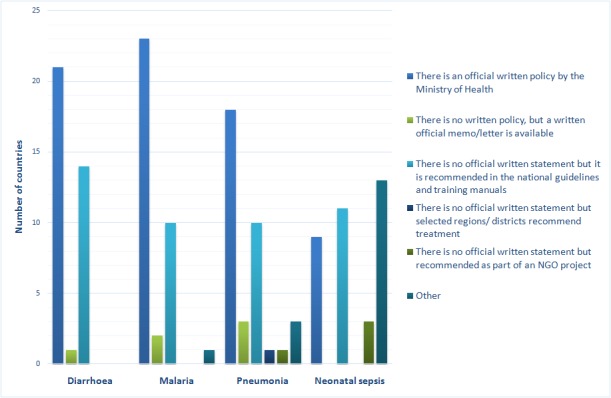
National policies on community case management in sub–Saharan Africa, by condition (n = 36).

Most countries had a national policy, memo or written guidelines for the implementation of CCM for diarrhoea (in 36 countries) and malaria (in 35 countries), with a slightly lower number for pneumonia (in 31 countries), as shown in **Figure 2**. Twenty countries now had a national policy, memo or written guidelines for neonatal sepsis.

### Institutional involvement in CCM

Ministries of Health, multilateral agencies and NGOs were all reported as having major roles in CCM activities within countries. In 35 countries, it was reported the Ministries of Health had a major role. 36 countries reported that UNICEF had a major role while the corresponding number of countries for the World Health Organization (WHO) was 34. National NGOs played a major role in 24 countries and international NGOs in 32 countries. Research institutions had a major function in CCM activities in 14 countries and private sector groups in 5 countries.

### Monitoring, supervision and reporting

Monitoring and supervision of CHWs who provide CCM services was provided by health facilities in 33 countries. Community supervisors performed this role in 14 countries, and health committees in 9 countries. Other mechanisms were used for supervision in 3 countries. Again, many countries employed more than one of these forms of supervision for CHWs.

CHWs also reported activities and patient data to health facilities in 33 countries and to community supervisors in 14 countries. In 3 countries, reporting was undertaken to health committees, while 4 countries also used other mechanisms. No countries reported an absence of a reporting function.

Thirteen countries reported the existence of a comprehensive national monitoring and evaluation plan for CCM activities including programme goals and objectives and indicators to be measured, with details of tools, frequency, and level of indicators, methodologies and dissemination. Thirteen countries reported a partial plan that covered only some of these components. In 9 countries, there was no monitoring and evaluation plan.

### Financing of CCM activities

Nine countries reported that there was a budget line in the domestic government budget for CCM. 24 countries reported that there was not.

**Figure 3:**
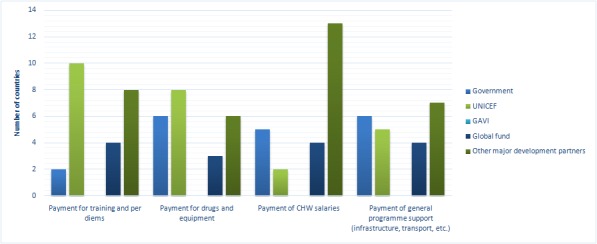
Institutions providing primary funding for different aspects of community case management in sub–Saharan Africa (n = 27). CHW – community health worker.

**Figure 3** presents data reported on the institutions providing primary funding for different aspects of CCM, including but not limited to the national government – overall, national governments were the primary funder for a minority of CCM programme components.

### Future plans

**Figure 4:**
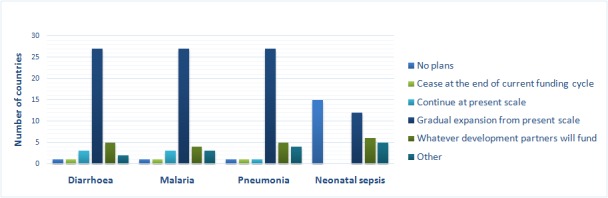
Future plans for community case management in sub–Saharan Africa, by condition (n = 42).

Most countries plan gradual expansion for existing CCM activities. However, many countries’ plans were dependent on what development partners will fund and a large group of countries had no plans for CCM for neonatal sepsis. **Figure 4** presents data on future plans for CCM activities for each of the conditions.

Fifteen countries reported that the government planned to increase their percentage of total funding for CCM services in their country. Fifteen countries reported not planning to do so, and 4 countries reported being in the process of developing a sustainable finance model.

## DISCUSSION

The results of the survey reported in this paper confirm that the scale of implementation of CCM by CHWs as a strategy to improve effective coverage of essential health services for children under–5 in sub–Saharan Africa has increased. The number of countries implementing CCM has risen since 2010 for each of the conditions, and overall, 28 countries in sub–Saharan Africa now report implementing CCM for pneumonia, diarrhoea and malaria, or “iCCM”.

The findings of the survey build on and confirm previous profiles of CHWs providing CCM services [11]. The diverse nature of CHW cadres involved in implementing CCM implies that a contextualised approach to how CCM dovetails with health systems strengthening is essential. The existence of thousands of CHWs implementing CCM provides greater opportunities to evaluate their effectiveness, building on existing knowledge, particularly to show that CCM at scale can make a meaningful contribution to ending preventable child deaths. There is also the possibility to better understand the strategic importance of CHWs in the context of the current global crisis in human resources for health.

The study provides several findings of note for national and global partners involved in the implementation of CCM. First, the persistence of user fees and mark–ups for CCM services in several countries requires attention. This occurs mostly in West Africa but given the evidence of the negative impacts of user fees, particularly on the poor, sustainable solutions must be developed as part of broader health system reform [13]. Discussions on CCM and CHWs need to inform global and national efforts to move towards universal health coverage. The slow uptake of the provision of salaries for CHWs should also be of concern for sustaining CCM programmes, notwithstanding that the debate on the benefits and adverse consequences of voluntarism has not been settled [14,15].

Second, despite the documentation of challenges in the development of CCM policy [16], most countries in sub–Saharan Africa have been able to develop at least some sort of written basis for CCM activities. The focus now must thus be on implementation of these policies and guidelines, including a greater focus on monitoring and evaluation of performance, quality and impact.

Third, financing and sustainability of CCM is a key issue – with CCM funding still largely driven by development partners, even for aspects that would be expected to be covered by governments, such as salaries and commodities. Only a minority of countries reported plans to increase the proportion of funding for CCM from domestic resources and few countries even have an item line for CCM activities in their domestic budgets. Further discussion of this important issue is presented in an accompanying paper [17].

Fourth, there is scope for expansion for newborn care. A small number of countries have started to embark on some CCM newborn activities. More support and guidelines are required from the global community to ensure this has a positive impact and further expansion occurs taking into consideration contextual factors such as CHW gender, existing workloads, and community and health system profiles. It is difficult to see how the goals of the Every Newborn Action Plan [18], to dramatically reduce newborn deaths, can be achieved without a greater role for community level engagement, including CHWs.

There are some limitations to this survey. It represents the expert opinion of the respondents. The extent to which this opinion was backed by data in national systems was dependent on its availability and at the discretion of the respondents, although triangulation was attempted through review of responses by regional experts. While surveys such as this provide useful data about trends in CCM policy and implementation, they are no substitute for improvements in national health management information systems, which must incorporate CCM activities and CHWs in general as integral parts of national health systems. For some questions in the survey (particularly around scale and cost), there were concerns about the completeness and quality of some of the reported data and hence these findings have not been included here. There seem to be generalised and significant knowledge gaps within many countries on the status of key aspects of CCM implementation, including, in some cases, basic information such as the number of CHWs that exist in the country.

Care must also be taken in interpreting the study findings with respect to scale of implementation. While 28 countries report implementing CCM for pneumonia, diarrhoea and malaria, the scale of implementation varies widely between these countries. This survey attempted to quantify the scale of implementation, but as noted above, data about the scale and cost of CCM activities is particularly lacking in many countries. Other instruments should be employed to provide an in–depth understanding of the scale of implementation of CCM activities, which is a crucial aspect to evaluate their potential success and impact, as well as to measure the quality of services.

In conclusion, this survey shows that much has been achieved in the development of policy and in the implementation of CCM to reduce child deaths in sub–Saharan Africa over the past decade. A major priority overall, discussed elsewhere, is the need to place CHWs and CCM as integral parts of national health systems [17]. Doing so is key to realizing the potential of CCM but also to addressing some of the information gaps on CCM activities revealed by this survey.
